# Karyotypic and Molecular Analysis of *Pterygoplichthys pardalis* (Castelnau 1855) from the Lower Amazon River

**DOI:** 10.3390/ani13091533

**Published:** 2023-05-03

**Authors:** Alcimara dos Santos Guimarães, Luan Aércio Melo Maciel, Mendelshon Fujiie Belém de Souza, Luís Reginaldo Ribeiro Rodrigues

**Affiliations:** 1 Graduate Program Natural Resources of Amazonia—PPGRNA, Federal University of Western Pará—UFOPA, Tapajós Campus, Vera Paz Street, Santarém 68040-255, PA, Brazil; alcimaraguimaraes@gmail.com; 2 Graduate Program Society, Nature and Development—PPGSND, Federal University of Western Pará—UFOPA, Tapajós Campus, Vera Paz Street, Santarém 68040-255, PA, Brazil; 3 Genetics and Biodiversity Laboratory—LGBio, Educational Sciences Institute—ICED, Federal University of Western Pará—UFOPA, Tapajós Campus, Vera Paz Street, Santarém 68040-255, PA, Brazil

**Keywords:** acari, exotic population, COI, karyotype, ornamental fish

## Abstract

**Simple Summary:**

This article discusses genetic traits (chromosomes, mtDNA) of the Amazon sailfin catfish. Domesticated fish of this species escaped from aquariums to establish invasive populations in large areas of North America and Asia. In Brazil, this species is an important fishery resource and a potential for fish farm management and production. Its karyotype showed 52 chromosomes with a similar conservative gross morphology to that of related species. The Cytochrome Oxidase I (COI) gene revealed a low molecular divergence between native and exotic populations.

**Abstract:**

*Pterygoplichthys pardalis* is an armored catfish native to South America and an important resource for the ornamental fish industry. Recently, several exotic populations have been introduced into rivers on five continents. Despite its commercial and environmental importance, *P. pardalis* is poorly studied from a genetic perspective. In this study, we analyzed the karyotype of *P. pardalis* from the Amazon River and molecular variations in the mitochondrial gene Cytochrome oxidase I (COI) between native and exotic populations. The karyotype presented diploid number 2n = 52 and NF = 100 without cytogenetic variation between males and females. Nucleolus organizer regions (Ag-NOR) in the distal region of the long arm of pair 12 coincided with the 18S hybridization signal, whereas 5S was syntenic to this chromosome but localized in the short arm. The constitutive heterochromatin was restricted in the distal regions of pairs 4, 12, 25, and 26. Telomeric probes showed only distal hybridization signals. The karyotype of *P. pardalis* diverged from that of its congeners, and COI molecular variation revealed four haplotypes. The Philippine population revealed the greatest diversity with three haplotypes, while haplotype H1 was the most abundant and observed in both native and exotic populations. This new genetic data contributes to species management and provides useful information from an aquaculture perspective.

## 1. Introduction

The Amazon basin is the largest hydrographical province in South America and supports considerable ichthyofauna with thousands of species. The armored catfish (Family Loricariidae, Siluriformes) is one of the main fish groups in this region and currently encompasses 1041 valid species [1].

The Loricariidae family is considered a monophyletic group; however, the systematic relationships at the subfamily level are unclear [2,3,4,5]. The sailfin plecos of the genus *Pterygoplichthys* (Gill, 1858) encompass 15 species [1]. *Pterygoplichthys* is closely related to *Hypostomus* and part of *Hemiancistrus*, which are nested in the clade–Tribe Hypostomini [2]. While the monophyly of Hypostomini is clear, there are some deviations, such as that of the taxonomic status of the “*Hemiancistrus*”, which was demonstrated to be a paraphyletic group [5]. The taxonomic issues in the Hypostomini clade also include the species delimitation in *Hypostomus* and *Pterygoplichthys*. For example, a recent integrative taxonomic study demonstrated that *Hypostomus chrysostictus* should be properly reclassified as *P. chrysostictus* [6].

Eight species of *Pterygoplichthys* have been recorded in the Amazon basin: *P. disjunctivus* (Weber 1991), *P. gibbiceps* (Kner 1854), *P. joselimaianus* (Weber 1991), *P. lituratus* (Kner 1854), *P. pardalis* (Castelnau 1855), *P. punctatus* (Kner 1854), *P. weberi* (Armbruster & Page 2006), and *P. xinguensis* (Weber 1991). *Pterygoplichthys pardalis* inhabits marginal lagoons and floodplains along the Amazon River and the confluence zones of its main tributaries [7]. *Pterygoplichthys pardalis* was possibly introduced into Asia and North America through the release or escape of animals sold in the aquarium market, that subsequently bioinvaded various river systems [8,9]. The exotic populations of *P. pardalis* reportedly caused severe ecological disturbances to native fish communities in Mexico and Southeast Asia [10,11].

In Brazil, *P. pardalis* is an important fishery resource that was considered one of the ten most exploited species in the lower Amazon region [12]. Moroni et al. [13] evaluated the biological features of *P. pardalis* for domestication and aquaculture programs. Those authors highlighted that the limited genetic data on *P. pardalis* was a constraint for its use in aquaculture production. Genetic information is necessary to achieve the management and conservation of commercial fish stocks, either in the wild or in fish farms [14,15,16].

Recent cytogenetic studies have contributed to the knowledge of fish genomes and karyotypic evolution. Cytogenetics is usually the first line of genetic information for taxonomy and evolutionary issues [17]. In addition, chromosomal markers are useful for aquaculture purposes, such as for polyploidy constructs [18,19], karyotype variation assessments [20], and hybrid monitoring [21]. Currently, seven species of *Pterygoplichthys* have been karyotyped (Table 1). Despite of conservative diploid number of 2n = 52, variations in chromosome morphology and C-bands were detected among species and populations (Table 1). The hypothetical ancestral karyotype of Loricariidae is assumed to have 2n = 54 [22], which implies a reduction of diploid number to 2n = 52 in the common ancestor of Hypostomini [2,23].

In this study, we introduced new cytogenetic data of *P. pardalis* from the lower Amazon River and performed a comparative karyotypic analysis of these with those of congeners and other Hypostomini taxa. We used the mitochondrial gene COI to explore genetic relationships between native and exotic populations of *P. pardalis.*

## 2. Materials and Methods

### 2.1. Sampling and Study Area

**Figure 1:**
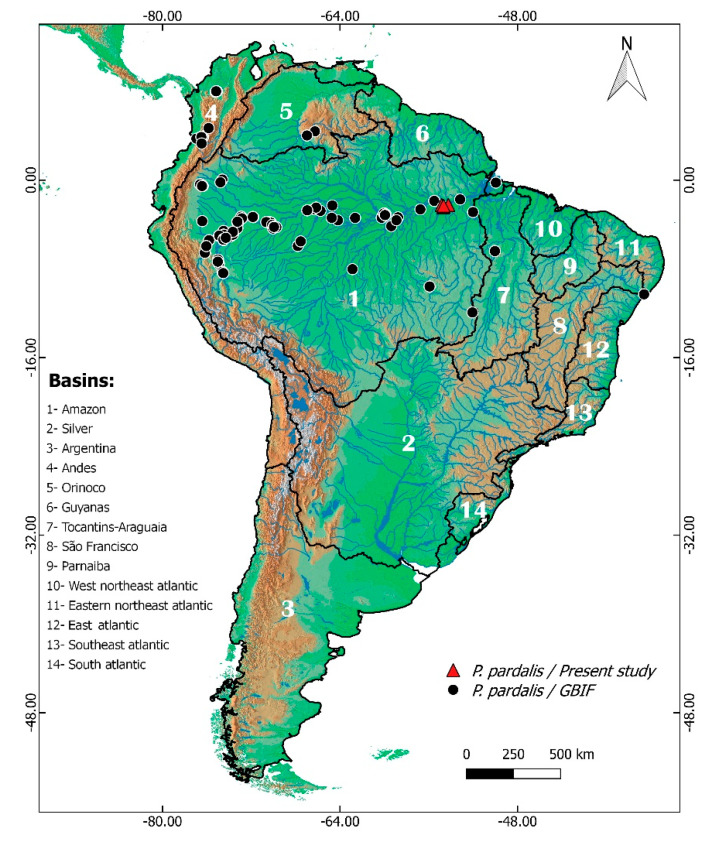
Distribution map of *Pterygoplichthys pardalis* along the South American hydrographic basins, which are outlined in black. The occurrence records (black circles) were compiled from the Global Biodiversity Information Facility (www.gbif.org, accessed on 17 April 2022).

This study included 10 females *Pterygoplichthys pardalis* individuals, 6 males and one specimen with unidentified sex (*n* = 17, Supplementary File Table S1). The fish were captured with gillnets by artisanal local fisherman, transported to the laboratory or field station for acclimation, and maintained with mitosis stimulation in 50 L plastic tanks filled with river water and aerated with aquarium pumps. The specimens were weighed, measured for standard length, and photographed, and muscle samples were collected for molecular analysis. The sampling sites were situated on the lower Amazon River in the municipality of Santarém, Pará State, Brazil. Four localities were sampled: (1) Porto dos Milagres, (2) Arapixuna, (3) Lago Grande, and (4) Lago Pacoval. A distribution map showing the occurrence of *P. pardalis* in South America is provided in Figure 1.

### 2.2. Ethical Statements

The fishes were anesthetized and euthanized with clove oil following a humane procedure approved by the Comitê de Ética em Pesquisa com Animais–CEUA/UFOPA (No. 1020180043). The voucher specimens were fixed with 10% formalin followed by preservation in 70% ethanol for deposit in the Fish Collection of the Federal University of Western Pará, collection numbers UFOPA-I 1393–UFOPA-I 1415. The collections were authorized by the Brazilian government under SISBIO permission No. 69419-1.

### 2.3. Chromosome Preparation and Cytogenetic Analysis

The preparation of mitotic chromosomes from the cephalic kidney involved 24 h of mitosis stimulation with yeast [30], followed by metaphase arrest with 0.0125% colchicine (0.01 mL/g body mass) for 30–40 min [22]. The tissue fragments were minced and incubated in hypotonic 0.075 M KCl solution for 20 min at 37 °C and fixed with fresh cold methanol-acetic acid fixative (3:1 *v*/*v*).

Air-dried slides were processed with 5% Giemsa conventional staining for karyotyping and the nucleolar organizing regions were detected using silver staining (Ag-NOR) [31]. C-banding was processed with barium hydroxide and 2 × SSC warm solutions [32] and stained with propidium iodide [33]. Fluorochromes DAPI (4′,6-Diamidine-2′-phenylindole dihydrochloride) and CMA3 (Chromomycin A3) were used to detect AT- or GC-rich sites [34,35], while FISH experiments were performed to detect telomeres and the rDNA cistrons 18S and 5S [36]. The probes were created by PCR using the following primers: Telomeres F (5′-TTAGGG-3′) and R (5′-CCCTAA-3′) [37]; 5SA (5′-TACGCCCGATCTCGTCCGATC-3′), 5SB (5′-CAGGCTGGTATGGCCGTAAGC-3′) [38]; 18S IpF (5′-CCGCTTTGGTGACTCTTGAT-3′), and 18S IpR (5′-CCGAGGACCTCACTAAACCA-3′) [39]. The PCR was assembled with a final volume of 25 μL as follows: 10.25 μL ultrapure water, 12.5 μL 2× mastermix (Fermentas), 0.5 μL of each primer (5 μM), 1 μL template DNA, and 0.25 μL Taq DNA polymerase (5 U/μL). No template was used for the telomere PCR and the PCR profile was Telomere–94 °C/5′, [94 °C/1′, 55 °C/30″, 72 °C/1′ × 10], [94 °C/1′, 60 °C/30″, 72 °C/1′30″ × 35] 72 °C/5′; DNAr5S–94 °C/1′, [95 °C/1′, 57 °C/1′, 72 °C/1′30″ × 35], 72 °C/5′; DNAr18S–95 °C/1′, [94 °C/1′, 56 °C/1′, 72 °C/1′30″ × 35], 72 °C/5′. The PCR products were inspected using 1% agarose gel stained with GelRed and positive reactions were labeled with biotin-14-dATP by nick translation using the Bionick Labeling System (Invitrogen, Waltham, MA, USA) or with digoxigenin using the DigNick system (Roche, Basel, Switzerland) following the manufacturers’ instructions. Detection washes followed with Avidin-FITC or Rodhamine-antidigoxigenin conjugates. The slides were counterstained with 2 mg/mL DAPI in Vectashield (Vector, Newark, CA, USA) mounting medium. Digital images were acquired using a Nikon EclipseCsl microscope (Melville, New York, NY, USA) coupled with a CCD camera and processed with Nikon NIS-Elements or Adobe Photoshop. All cytogenetic analyses and karyotype determinations were captured with a minimum of 10 metaphases per experiment.

### 2.4. Molecular Methods

Genomic DNA of six *P. pardalis* specimens (PML-27, PML-30, APX-01, APX-02, APX-04, and APX-05) was extracted following the salting-out protocol [40]. The 5′ region of the mitochondrial gene Cytochrome oxidase c subunit I (COI) was amplified by PCR using DNA barcoding with the standard primers FishF1 and FishR1 [41]. The reactions were assembled to a 25 μL final volume as follows: 15 μL of ultrapure water, 2.5 μL 10× buffer (200 mM Tris-HCl pH 8.4 + 500 mM KCl), 2.5 μL of 50 mM MgCl_2_, 2.8 μL of 1.25 mM DNTPs, 0.5 μL of 5 μM for each primer, 0.2 μL of Taq DNA polymerase (5U/μL), and 1 μL of genomic DNA (50–100 ng/μL). The cycling profile was 95 °C/2′, 35 cycles of 94 °C/30″, 54 °C/30″, and 72 °C/60″ followed by a final extension of 72 °C/10′. The PCR products were visualized in 1% agarose gel stained with GelRed^TM^ (Biotium Inc., Fremont, CA, USA).

Positive reactions were cleaned with 20% PEG 8000 [42] and processed for capillary sequencing using the ABI PRISM BigDye Terminator V.3 Cycle Sequencing kit (Applied Biosystems Inc., Foster city, CA, USA) with the genetic analyzer ABI3500, following the manufacturer’s instructions.

The COI sequences were visually inspected and trimmed with BioEdit v.7.2.5 [43]. We checked the species taxonomic identity of the sequences using a nucleotideBLAST search (https://blast.ncbi.nlm.nih.gov/Blast.cgi, accessed on 2 March 2022) and an ID engine tool (http://boldsystems.org/index.php/IDS_OpenIdEngine, accessed on 3 March 2022). Supplementary sequences of *P. pardalis* and other Hypostomini members were downloaded from GenBank (https://www.ncbi.nlm.nih.gov/) (Supplementary File Table S2). For molecular analysis, we assembled sequences of *P. pardalis* (*n* = 80), including specimens collected from natural populations (Brazil and Colombia) and exotic populations from Asia, representative of six countries. In addition, *P. edentaculatus* (*n* = 5), *P. zuliaensis* (*n* = 1), and *Hypostomus cochliodon* (*n* = 1) were used as outgroups. The best model for the data was that of T92+G (Tamura 3-parameter) and the gamma shape parameter was 0.63. A distance matrix and neighbor-joining clustering were generated with MEGA X [44]. Statistical support was evaluated from 1000 bootstrapping iterations. The tree topology/design was edited with FigTree v.1.4.4 [45].

To explore the molecular variation and microevolutionary relationships within natural and exotic *P. pardalis* populations, we created a haplotype network using the median joining algorithm [46]. Haplotypic data were obtained using DNAsp v.5.10.01 [47] and the network drawing was processed with Network 10.2.0.0 (Fluxus Technology Ltd., Colchester, Essex, England).

## 3. Results

### 3.1. Cytogenetic Analysis

**Figure 2:**
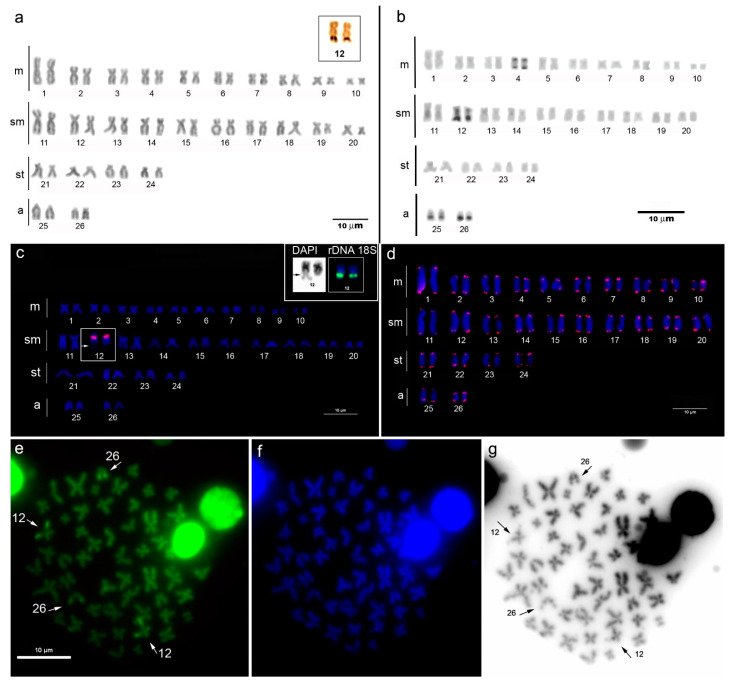
Karyotypic and cytogenetic markers of *Pterygoplichthys pardalis* from the lower Amazon River. (**a**) Giemsa conventional staining and Ag-NOR marks on pair 12 (inset box); (**b**) C-banded karyotype; (**c**) karyotype showing the 5S rDNA hybridization (red marks) and a conspicuous secondary constriction co-localized with 18S rDNA (green marks) on pair 12 (inset box); (**d**) karyotype showing telomere probe hybridization (red marks); (**e**,**f**) CMA3-DAPI stained metaphase showing GC rich sites on chromosomes 12 and 26; (**g**) image of inverted DAPI metaphase showing a secondary constriction co-localized with the GC rich site on chromosome 12.

The karyotype of *P. pardalis* from the lower Amazon River has 2n = 52 chromosomes and an FN = 100, a karyotypic formula of 20m + 20sm + 8st + 4a, and no distinction of differentiated sex chromosomes (Figure 2a). The Ag-NOR staining revealed one pair of NORs in at the subtelomeric region of the long arm of pair 12 (Figure 2a, inset box). Constitutive heterochromatin was detected in narrow bands only in four chromosome pairs, and it was bitelomeric in pair 4 and distal in pairs 12, 25, and 26 (Figure 2b). Ribosomal gene 5S was detected in the centromeric region of the submetacentric pair 12 (Figure 2c). This chromosome also showed hybridization of the rDNA 18S probe in the subtelomeric region of the long arm, where it was coincident with a secondary constriction (Figure 2c, inset box). The telomere motifs (TTAGGG) were marked only in the chromosome tips, with no evidence of interstitial telomere sequences (Figure 2d). CMA3-DAPI double staining revealed brilliant GC-rich sites in two chromosome pairs, of which one was identified as pair 12, because it showed a conspicuous secondary constriction that was co-localized with the Ag-NOR, 18S and CMA3 marks in the same position as that of the NOR and rDNA 18S, while the second pair was the acrocentric pair 26 (Figure 2e–g).

### 3.2. Molecular Analysis

**Figure 3:**
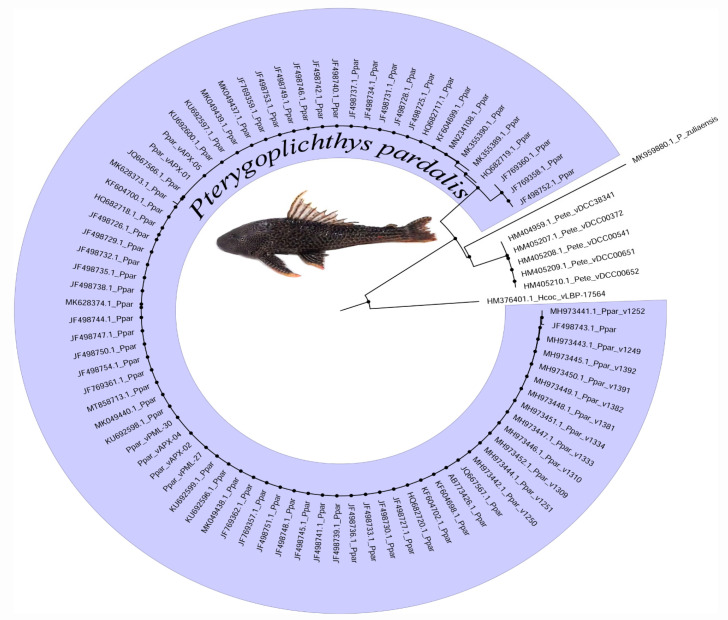
Neighbor-joining clustering of *Pterygoplichthys* based on COI nucleotide variation. The nodes of *P. pardalis* are shaded in blue. *Hypostomus cochliodon* was adopted as an outgroup.

**Figure 4:**
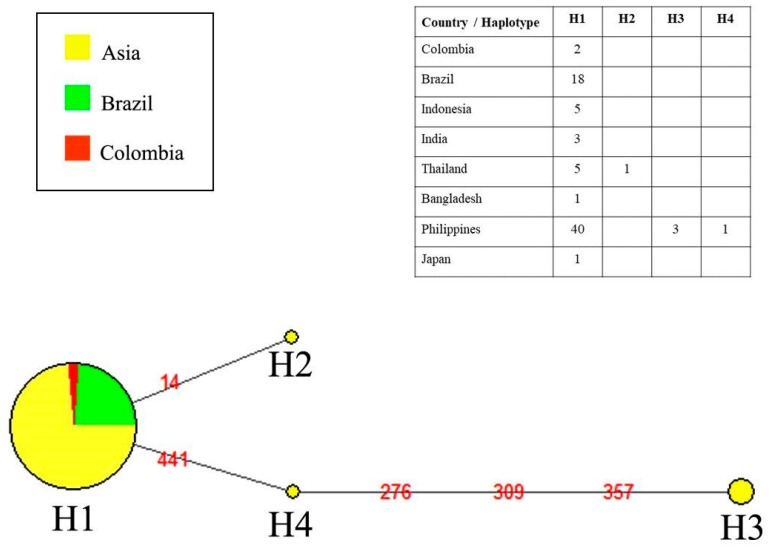
Median-joining haplotype network of COI sequences from natural and exotic populations of *Pterygoplichthys pardalis*. The embedded table (top right) shows the haplotype frequency by country. The mutational steps are indicated by the nucleotide site positions (red numbers on the lines).

The COI sequence of fish samples from the lower Amazon River revealed a 100% taxonomic identity with that of *P. pardalis* reference sequences from GenBank. Our assembled COI dataset alignment included 80 sequences of 591 bp in length, with the exception of a few individuals that had shorter sequences. The sequence matched the positions 5581 through 6172 (5′–3′) in the *P. pardalis* reference mitogenome (Access Number NC_060468.1). G+C content was 0.421, polymorphic sites were S = 5, and nucleotide diversity was Pi = 0.00071. The genetic distance within *P. pardalis* populations was zero (Table 2), which shows an extensive genetic homogeneity of this species to the COI locus. In contrast, we found clear discriminations between *P. pardalis* and *P. zuliaensis* (0.071 to 0.085) and *P. etentaculatus* (0.030 to 0.031). The neighbor-joining distance-clustering tree is shown in Figure 3. In this study we found four COI haplotypes (h = 4) of *P. pardalis* (*n* = 80) that were representative of indigenous and exotic populations, and the haplotypic diversity was Hd = 0.121. All COI variants were observed in the Asian populations, whereas the native populations from Brazil and Colombia had only a single haplotype (H1). This haplotype was the most frequent and shared by both native (Brazil and Colombia) and exotic populations (Asia). The haplotype H2 was a singleton recorded from a specimen collected in Thailand, while H3 and H4 were recorded in specimens from the Philippines. Both H2 and H4 haplotypes diverged from H1 by one mutation, whereas H3 diverged by four mutational steps (Figure 4).

## 4. Discussion

*Pterygoplichthys pardalis* specimens from the lower Amazon River (Santarém sector) showed extensive conservative patterns of karyotype features and chromosome morphologies, as previously observed in the literature [24,25,26,27,28,29]. However, we found a minor divergence in chromosomal morphology between this population and that of *P. pardalis* from the Manaus section of the Amazon River [29]. This difference was demonstrated by a modification in the karyotypic formula: 20m + 20sm + 8st + 4a in the population from Santarém and 18m + 18sm + 8st + 8a in *P. pardalis* from Manaus [29]. This cytogenetic variation may have resulted from pericentric inversion rearrangements that transformed two pairs of bi-armed chromosomes into acrocentrics, or vice versa.

Karyotypic evolution in freshwater fish is linked to species biological traits (e.g., deme size, migration behavior) and environmental structures that could restrain gene flow between populations, thereby favoring the fixation of chromosomal rearrangements [48]. Despite the dispersion of *P. pardalis* populations throughout the main channel of the Amazon River without topographic barriers that promote isolation, this species is a non-migratory fish that is limited to a local distribution [49]. Moreover, the watercourse in the section of the Amazon River between the cities of Manaus and Santarém measures approximately 716 km and receives discharge from large tributaries (e.g., Trombetas, Nhamundá, Madeira, and Negro rivers) that potentially affects the water chemistry in the confluence zones. This can induce local adaptive demands that hypothetically influence the dispersal of the population. Therefore, it is plausible that *P. pardalis* has a karyotypic polymorphism along its natural geographic range. This mechanism of chromosomal evolution in not uncommon in the genetic divergence of Amazon fishes. In addition, populations of *Peckoltia vittata* (Loricariidae, Hypostominae) from the Xingu River diverged in terms of a variation in chromosome arms, which was explained by pericentric inversions [50].

Furthermore, the chromosomal morphology of *P. pardalis* diverged from *P. multiradiatus, P. anisitsi,* and *P. joselimaianus* (see Table 1), which suggests that pericentric inversion and/or heterochromatic addition rearrangements acted in the karyotypic evolution of the group. However, the full spectrum of chromosome variation has been biased by visual interpretations of chromosome morphologies based on arm ratios. Tiny chromosomes and over condensed metaphases can cause misinterpretations of chromosome morphologies. A focus in *Pterygoplichthys* is the number of acrocentrics, which range from zero (*P. multiradiatus, P. gibbiceps, and P. joselimaianus*) to 16 (*P. anisitsi* from the Miranda River) [24]. The literature review showed a clear improvement in terms of chromosome quality in the most recent studies and the unequivocal discrimination of acrocentric pairs, which has been improved through the use of banding and FISH markers. Therefore, the acrocentric threshold counts of zero and 16 are likely due to poor-quality chromosome preparations and/or limited cytogenetic markers. The smallest chromosome of *P. pardalis* (pair 26) is acrocentric and shows a positive C-band subterminal, that is characterized as GC-rich heterochromatin (Figure 2b,e). This combination of cytogenetic markers is recommended for further studies on karyotypic evolution in *Pterygoplichthys*.

Heterochromatin (C-bands) was rare in the *P. pardalis* specimens from Santarém, which contrasted with the large number observed in the population from Manaus [29]. Araújo da Silva et al. (2019) argued that the C-banding pattern may be associated with water pollution and that *P. pardalis* shows heterochromatic magnification when exposed to a polluted environment. The collection site of *P. pardalis* from the lower Amazon River in the present study was assumed to be an unpolluted environment, because it was distant from urban zones and other potential sources of water pollution, such as large-scale agriculture, mining, and industry. The heterochromatic C-bands in *P. pardalis* from Catalão Lake, an unpolluted site from the Manaus sector, showed marks in the centromeric regions of all chromosomes [29]. Therefore, it appears that *P. pardalis* has an intrinsic C-banding polymorphism that is not exclusively associated with genomic adaptation to environmental disturbances. The mechanisms involved in the heterochromatin organization of fish genomes are not completely understood; however, it is clear that heterochromatin accumulation can play a role in sex chromosome evolution [51] and speciation [52].

Karyotypes with a single Ag-NOR-bearing chromosome are considered plesiomorphic in many fish groups, including the primitive Actinopterygii [53]. This pattern is shared by the *Pterygoplichthys* species investigated to date and is considered to exist in the ancestral Loricariidae karyotype [54,55,56]. Despite the conservatism of the number (two Ag-NORs), the *Pterygoplichthys* karyotypes showed a size heteromorphism and a preferential subterminal position in submetacentric (4 sp), metacentric (2 sp), and subtelocentric 1 sp; *P. chrysostictus*) pairs.

The Ag-NOR labeling detected by silver nitrate staining was highly associated with hybridization signals of the 18S rDNA probe [53]. Three species of *Pterygoplichthys* (*P. ambrosettii*, *P. multiradiatus,* and *P. pardalis*) showed coincidental Ag-NOR and 18S FISH signals (see Table 1). Ag-NOR is located subterminally on the long arm of a submetacentric chromosome in *P. pardalis* and *P. ambrosettii,* while in *P. multiradiatus* Ag-NOR is subterminal on a metacentric chromosome. Homology between these Ag-NOR-bearing chromosomes implies that pericentric inversion shifted the centromere position but preserved Ag-NOR/18S. The morphology of Ag-NOR-bearing chromosomes changed in *P. chrysostictus* (subtelocentric) and *P. joselimaianus* (metacentric), although the subterminal location of Ag-NOR was preserved. Similarly, 5S rDNA cistrons were detected in synteny with 18S in *P. ambrosettii* and *P. pardalis* (see Table 1). In this study, we evidenced rDNA 5S/18S synteny in *P. pardalis* from Santarém, due the observation of a conspicuous secondary constriction subterminal in 12q, which showed the 5S hybridization mark in the pericentromeric region. Secondary constriction is correlated with the NOR position in a chromosome and indicates the site of rDNA 18S. This 5S/18S rDNA syntenic configuration is interpreted as a plesiomorphic trait in Loricariidae [56]. *Pterygoplichthys* retain conservative cytogenetic traits (2n = 52, single Ag-NOR) that are shared with *Hemiancistrus* [43] and *Aphanotorulus emarginatus* (*Hypostomus emarginatus*) [56]. However, *Hypostomus* is cytogenetically diverse with karyotypes having diploid numbers ranging from 2n = 64 to 2n = 84 [23]. Therefore, the primitive status of *Pterygoplichthys* in the Hypostomini phylogeny is well supported cytogenically.

DNA barcoding (COI) was effective for identifying *P. pardalis* and revealing its considerable divergence (3–8.5%) from its congeners (*P. etentaculatus* and *P. zuliaensis*). Molecular species delimitation by DNA barcoding has been successfully used for monitoring biodiversity and addressing taxonomic ambiguities, especially in commercially exploited species [57]. Considering that the taxonomy of *Pterygoplichthys* is controversial and that the delimitation of some species of the genus is based on morphological characters that are not very robust, such as the pattern of ventral spots [58], the COI gene can be useful in the reconstruction phylogenetics of this genus. However, in exotic populations of *P. pardalis* and *P. disjunctivus*, molecular identification by DNA barcoding and morphological patterns is limited due to possible interspecific hybridization [58,59,60,61]. The use of cytogenetic markers associated with DNA barcoding and morphological analysis can be an effective approach to assist in resolving the taxonomy of the group and investigating the dynamics of introgression in exotic populations.

Populations of introduced species commonly show distinct losses of genetic diversity associated with the colonization process, due to the founder effect and genetic drift [62]. However, the process of adapting to a new environment can result in population growth and dispersion to new areas of the colonized environment [62]. Invasive populations of *P. pardalis* have established themselves in Mexico [11] and several Southeast Asian countries [8]. We found a low genetic variation in *P. pardalis* COI sequences within the native range (Brazil), with only a single mitochondrial haplotype (COI) detected in *P. pardalis* from the Amazon River (Manaus sector) [29,57]. This same haplotype (H1), annotated as P1 by Wu et al. (2011) [63], was recorded in the lower Amazon River population in the current study and is widely dispersed in exotic populations in Mexico and Asia (Bangladesh, Philippines, India, Indonesia, Japan, and Thailand [58,59,60,61]. In Asia, exotic populations of *P. pardalis* exhibit high morphological and genetic diversity, which has been suggested to result from interspecific hybridization between *P. pardalis* and *P. disjunctivus* [59,60]. We observed three haplotypes in the exotic population of the Philippines, one of which (H1) was shared with native populations, which indicates that the Philippines may have been the origin of the colonization in Southeast Asia, or that this genetic variability in the Philippines resulted from multiple introduction events. The origin of *P. pardalis* in the Philippines remains unclear, but it is suspected that the founder individuals were imported from the United States through international trade in ornamental fish for domestic aquariums [10].

The natural geographic distribution of *P. pardalis* extends over a wide region of northern South America, including the Amazon and Orinoco River basins. To date, DNA barcoding has been used in only two locations in this region, the middle (Manaus) and lower Amazon River (Santarém), which limits or prevents a reliable assessment of the genetic diversity of the native population. Despite this scarcity of genetic data, *P. pardalis* is a heavily exploited fishing resource in the Amazon basin, both for subsistence fishing for traditional populations and commercial fishing to supply regional urban centers [63,64]. Santarém city is the main fishing port in the lower Amazon region and local fishing statistics reveal high heterogeneity in the composition and contribution of exploited species, including between different communities of small-scale artisanal fishers [65]. *P. pardalis* is considered the most important fishing resource for traditional riverside communities in the floodplain region of Santarém. In communities where this species is often captured, a decrease in average body size of the fish has been observed [66,67]. Further studies on population genetics of *P. pardalis* from the Amazon basin are recommended to evaluate the possible effects of overfishing and loss of intraspecific genetic diversity.

Construction of genetic databases are mandatory to subsidize technological packages for domestication and aquaculture programs. The sailfin catfish (*P. pardalis*) is a recognized aquaculture candidate species; however, the lack of genetic data is a main limiting factor and a high priority for research aimed at the domestication of this species for cultivation in aquaculture systems [13]. This new data on the karyotypic patterns and DNA barcoding (COI) sequences of *P. pardalis* from the wild population contribute to advances in the debate on Loricariidae taxonomy, the management of invasive species, and considerations regarding fishery pressures, stock conservation, and food security in the context of the sustainability of traditional Amazon riverside communities.

## 5. Conclusions

The *Pterygoplichthys pardalis* from the lower Amazon region retain the diploid number 2n = 52 but differ from congeners due to variations in the karyotypic formula. Pericentric inversion-type rearrangements may explain the changes in chromosomal morphology observed in the group. COI gene variation revealed that native and exotic populations of *P. pardalis* are highly homogeneous and linked by a single shared haplotype.

## Figures and Tables

**Table 1:** Summary of *Pterygoplichthys* cytogenetic data. Karyotypic formula (KF), C-banding (BC), Nucleolar organizing regions (NOR), Chromomycin A3 (CMA_3_), FISH of rDNA probes (18S and 5S), FISH of retrotransposons probes (Rex1, Rex3, Rex6), metacentric (m), submetacentric (sm), subtelocentric (st), acrocentric (a), not analyzed (-), analyzed (+).

Species	Locality	KF (m + sm + st + a)	BC	NOR	CMA3	18S	5S	Rex1	Rex3	Rex6	Authors
*P. anisitsi*	rio Tietê	28 + 12 + 8 + 4	-	16	-	-	-	-	-	-	[24]
*P. anisitsi*	rio Miranda	8 + 14 + 14 + 16	-	5	-	-	-	-	-	-	[24]
*P. anisitsi*	rio Iguatemi	14 + 26 + 8 + 4	+	9	9, 10	-	-	-	-	-	[25]
*P. anisitsi*	rio Preto	16 + 24 + 8 + 4	+	14	-	-	-	-	-	-	[26]
*P. ambrosettii*	rio Paraná	16 + 24 + 8 + 4	-	-	-	14	14	-	-	-	[23]
*P. multiradiatus*	rio Orinoco	22 + 18 + 12 + 0	-	10	-	10	-	-	-	-	[24,27]
*P. gibbiceps*	rio Orinoco	20 + 24 + 8 + 0	-	21	-	-	-	-	-	-	[24]
*P. joselimaianus*	rio Araguaia	28 + 16 + 8 + 0	-	9	-	-	-	-	-	-	[28]
*P. pardalis*	rio Amazonas	18 + 18 + 8 + 8	-	11	-	11	11	+	+	+	[29]

**Table 2:** Genetic distances based on COI sequence variations between *Pterygoplichthys* and *Hypostomus cochliodon*, a related taxa. The Tamura 3-parameter model was applied for distance estimates.

		1	2	3	4	5	6
1	*Hypostomus cochliodon*						
2	*Pterygoplichthys etentaculatus*	0.078					
3	*P. pardalis* (Asia)	0.086	0.031				
4	*P. pardalis* (Colombia)	0.084	0.030	0.000			
5	*P. pardalis* (Manaus, Brazil)	0.089	0.030	0.000	0.000		
6	*P. pardalis* (Santarém, Brazil)	0.086	0.031	0.000	0.000	0.000	
7	*P. zuliaensis*	0.127	0.068	0.075	0.071	0.085	0.075

## Data Availability

Supporting Data are included in the article manuscript and Supplementary Files.

## Supplementary Materials

The following supporting information can be downloaded at: https://www.mdpi.com/article/10.3390/ani13091533/s1, Table S1: Data of *Pterygoplichthys pardalis* (Castelnau 1855) collected from the lower Amazon River included in this study; Table S2: COI sequence metadata for *Pterygoplichthys* and *Hypostomus cochliodon* included in this study.
